# Associations of technostressors at work with burnout symptoms and chronic low-grade inflammation: a cross-sectional analysis in hospital employees

**DOI:** 10.1007/s00420-023-01967-8

**Published:** 2023-05-06

**Authors:** Helena C. Kaltenegger, Linda Becker, Nicolas Rohleder, Dennis Nowak, Caroline Quartucci, Matthias Weigl

**Affiliations:** 1 Institute and Clinic for Occupational, Social and Environmental Medicine, University Hospital, LMU Munich, Ziemssenstr. 1, 80336 Munich, Germany; 2 Institute of Psychology, Friedrich-Alexander-Universität Erlangen-Nürnberg, Erlangen, Germany; 3 Bavarian Health and Food Safety Authority, Institute for Occupational Health and Product Safety, Environmental Health, Munich, Germany; 4 Institute for Patient Safety, University Hospital Bonn, Bonn, Germany

**Keywords:** Burnout, C-reactive protein, Inflammation, Stress, Technostress, Work

## Abstract

**Objective:**

Despite the increasing scholarly interest in the phenomenon *technostress, *associated biological effects on employee health are under-researched. Chronic low-grade inflammation is suggested as a central pathway linking stress experience to disease development. The aim of this study was to assess associations of technology-related work stressors (technostressors) with low-grade inflammation and burnout symptoms.

**Methods:**

*N* = 173 (74.6% women, *M*_age_ = 31.0 years) university hospital employees participated in a cross-sectional study. Self-report questionnaires were used for the assessment of general psychosocial working conditions (work overload, job control, social climate), a range of different technostressors, burnout symptoms, and relevant confounders. Participants provided capillary blood samples, and high-sensitivity C-reactive protein (hs-CRP) as an inflammatory biomarker was analyzed from dried blood spots.

**Results:**

Based on a factor analysis, we identified four underlying dimensions of technostressors: techno- and information overload, techno-complexity, interruptions and multitasking as well as usability and technical support. In multivariate linear regressions, techno-/information overload and techno-complexity were associated with core (exhaustion, mental distance) and secondary (psychosomatic complaints) symptoms of burnout. Techno-/information overload was a significant predictor of burnout core symptoms, even when general work overload was controlled for. The technostressors were not associated with hs-CRP.

**Conclusion:**

This is the first study on technology-related stress at work and chronic low-grade inflammation. The results suggest that (information) overload caused by digital technology use is a distinct work stressor with genuine consequences for psychological health. To what extent these effects also manifest on a physiological level needs to be subjected to future studies, ideally with prospective designs.

**Supplementary Information:**

The online version contains supplementary material available at 10.1007/s00420-023-01967-8.

## Introduction

The advancing digitalization has pervasive consequences on the psychosocial work environment and thereby on workers’ health and well-being (Dragano and Lunau 2020; Parker and Grote 2022). These can be negative in terms of stress experience and impaired mental health, but also positive for workers’ health and well-being for instance due to greater flexibility in work organization, better access to information, or automation (Dragano and Lunau 2020; La Torre et al. 2019). Especially in healthcare, there have been fundamental advancements in terms of health information technology (e.g., electronic health records, computerized decision support systems; Abbott and Weinger 2020). At the same time, healthcare professionals are already exposed to a high degree of work stress putting them at an increased risk for adverse health outcomes (Dawe et al. 2016; Adriaenssens et al. 2015; Kaltenegger et al. 2022).

Introduced by Brod in 1982, the definition of the phenomenon *technostress* changed over time with the latest referring to “stress experienced by end users of Information and Communication Technologies (ICTs)” (Ragu-Nathan et al. 2008, p. 417). Tarafdar et al. (2007) compiled technology-related factors that can cause technostress (*techno-overload*, *techno-invasion*, *techno-complexity*, *techno-insecurity*, *techno-uncertainty*), i.e., so-called technostressors (La Torre et al. 2019). Further technostressors include work interruptions by ICTs (Galluch et al. 2015; Ninaus et al. 2015), multitasking (Reinecke et al. 2017), or information overload (Eppler and Mengis 2004; Tarafdar et al. 2007). Existing reviews on technostress report strain reactions in employees related to psychological (e.g., burnout, exhaustion), physiological (e.g., activation of stress hormones), cognitive (e.g., concentration problems) and behavioral (e.g., job performance) symptoms (La Torre et al. 2019; Dragano and Lunau 2020; Berg-Beckhoff et al. 2017; Riedl 2012; Borle et al. 2021). However, these reviews also reveal that research on the health consequences of technostress is still fragmented and the evidence base is limited. In particular, the following knowledge gaps remain:

First, while it is well-researched that exposure to workplace stressors is associated with mental health problems (Madsen et al. 2017; Aronsson et al. 2017), studies on work stressors related to digital technologies and mental health outcomes are sparse with first results suggesting associations with burnout (Dragano and Lunau 2020). Burnout is defined as “a work-related state of exhaustion that occurs among employees, which is characterized by extreme tiredness, reduced ability to regulate cognitive and emotional processes, and mental distancing. These four core dimensions of burnout are accompanied by depressed mood as well as by non-specific psychological and psychosomatic complaints” (Schaufeli et al. 2020, p. 4). The few studies on technostress and burnout were predominantly based on office workers (Berg-Beckhoff et al. 2017). However, burnout is of critical concern especially in clinical work with implications not only for staffs’ health but also for patient care and the entire healthcare system (Dall'Ora et al. 2020; West et al. 2018; Weigl 2022). Recent research in health professionals across different settings showed high to moderate levels of technostress and considerable associations with burnout symptoms amongst other health-related consequences (Golz et al. 2021; Kasemy et al. 2022). Specifically for electronic health record systems, current research among US physicians found that the usability was rated as poor and in turn, that perceived usability was related to provider task load and burnout with task load functioning as a mediator (Melnick et al. 2020a, b). Thus, more investigations on technostress and burnout in healthcare workers are needed.

Second, technostress has mostly been assessed with self-report questionnaires, while objectively measurable biological effects have largely been overlooked. Few studies suggest that technostressors activate physiological stress responses. This was shown for the sympathetic nervous system as one domain of the autonomic nervous system (ANS; e.g., Galluch et al. 2015) and the hypothalamic-pituitary-adrenocortical (HPA) axis (Riedl et al. 2012; Arnetz and Berg 1996; Kasemy et al. 2022). However, these findings relate to *acute* stress rather than to the long-term effects of *chronic* stress. As in modern digitalized work environments, technostressors may occur recurrently over prolonged periods, they might lead to chronic stress experience (Day et al. 2010). The human stress response includes—beyond the activation of the main stress systems (ANS and HPA axis)—complex effects of the immune system, most importantly up-regulation of inflammatory pathways (Ulrich-Lai and Herman 2009; Segerstrom and Miller 2004; Morey et al. 2015; Chrousos 2009). In the short-term, these changes are critical for survival, however, in the long-term, wear-and-tear effects of the stress systems can occur (cf. allostatic load model (McEwen 1998; McEwen and Stellar 1993))—as for instance the phenomenon of chronic systemic low-grade inflammation. Low-grade inflammation is suggested as a central pathophysiological mechanism in the development of chronic conditions encompassing cardiovascular, metabolic, and neurodegenerative diseases, depression as well as cancer (Couzin-Frankel 2010; Liu et al. 2017). It is usually assessed by measuring concentrations of the acute phase protein C-reactive protein (CRP) or of cytokines (such as interleukins) in blood or saliva (Rohleder 2019). Adverse psychosocial factors at work were associated with low-grade inflammation in employees, yet with limited evidence (Kaltenegger et al. 2021; Wright et al. 2020). For a better understanding of the long-term psychophysiological health effects of technostressors, it is essential to assess biomarkers indicative of biological alterations of the stress systems, such as chronic low-grade inflammation.

Third, it is unclear whether technostressors are genuinely new, distinct stressors or if they are just antecedents or specific forms of other general psychosocial work stressors like work overload or job insecurity (Dragano and Lunau 2020). Therefore, it is crucial to investigate technostressors in their interplay with other job characteristics and to test for individual as well as interactive effects. For example, technostress in terms of a system breakdown in a human–computer interaction task only increased the skin conductance of male participants if they were under time pressure (Riedl et al. 2013).

As a theoretical foundation, we use the well-established job demand-control(-support) (JDCS) model (Karasek 1979; Johnson and Hall 1988; Johnson et al. 1989): It proposes that the combination of high job demands, low job control, and low social support at work leads to mental strain, which is linked to cardiovascular disease (CVD) morbidity and mortality. Furthermore, we draw on the challenge-hindrance stressor framework (Cavanaugh et al. 2000; LePine et al. 2005; Podsakoff et al. 2007) that has been applied to the technostress concept (Califf and Sarker 2020; Tarafdar et al. 2019): Based on the notion of a duality of negative and positive sides of technology, technostressors can be divided into *hindrance technostressors* and *challenge technostressors*. Hindrance technostressors are technology characteristics appraised by the user as disturbing or threatening and comprise the aforementioned technostressors; challenge technostressors in contrast, are appraised as promoting task accomplishment and hence, alleviate technostress (Tarafdar et al. 2019; Califf and Sarker 2020). Several challenge technostressors have been proposed in the literature, such as *technical support provision* by solving users’ ICT problems (Ragu-Nathan et al. 2008), and usability features consisting of *usefulness*, i.e., the degree to which technology improves job performance, as well as *reliability*, i.e., consistency and dependability of technology (Ayyagari et al. 2011).

In sum, we are only at the beginning of understanding the psychophysiological effects of technology-related stress at work—the research base is limited and there is a striking lack of studies on inflammatory (re-)activity as a major pathway in the transition to disease (Becker et al. 2022a, b; Kaltenegger et al. 2021). Therefore, this study sought to investigate associations of different risk factors at work, including technostressors and general psychosocial working conditions (job demands, control, social support), with burnout symptoms and low-grade inflammation among employees of a university hospital. In particular, we examined the following research questions:

1. Are technostressors associated with burnout symptoms?
2. Are technostressors associated with low-grade inflammation?
3. If associations in (1) and (2) are significant, (3a) are they also existent, when controlling for general psychosocial work factors? (3b) are associations moderated by other technostressors or by general work factors (i.e., interaction effects)?

## Methods

### Design and ethics

This cross-sectional analysis is based on data collected in 2021 (June-November) as part of a larger cohort study on work stress and health sequelae in employees of the University Hospital of Ludwig-Maximilian University (LMU) Munich, Germany. The study protocol has been registered (for more information see: https://osf.io/94p6n/). The study was approved by the Ethics Committee at the Medical Faculty of LMU (20–0914) and is being performed in accordance with the ethical standards of the 1964 Helsinki Declaration. All participants included in the study provided written informed consent.

### Participants

Persons undergoing an obligatory pre-employment medical examination at the Outpatient Clinic for Occupational, Social, and Environmental Medicine were invited to participate in the study. The sample thus consists of new employees at LMU University Hospital with different kinds of professions including nurses, physicians, (medical-) technical, research and administrative staff, etc. Prior to inclusion, participants received information concerning study objectives and procedures. Data collection took place on-site at the outpatient clinic in medical examination rooms. For this study, a subsample of *N* = 173 (74.6% women, *M*_age_ = 31.0 years) was analyzed consisting of participants who had already started their job or who had not started at that time, but who had been employed prior to the beginning of their employment at LMU University Hospital. The following eligibility criteria were applied: Persons with a temporary contract of less than six months were not included. Furthermore, persons reporting current symptoms indicating acute infection or inflammation (such as acute cold, fever, acute injuries, cystitis, etc.), permanent anti-inflammatory medication intake, recent intake of anti-coagulant drugs (last 12 h before testing), pregnancy, or insufficient German language knowledge were excluded. Participants with CRP levels > 10 mg/L were discarded a posteriori since concentrations above this cut-off suggest a medical source of infection or inflammation, what may bias the prediction of low-grade inflammation (Pearson et al. 2003).

### Measures

#### Predictors

##### General psychosocial work factors

A comprehensive questionnaire was developed for participants’ self-report of their individual work situation. In line with the JDCS model, it included three scales for the assessment of psychosocial working conditions derived from a well-established tool for work analysis (Glaser et al. 2020): *Work overload* was measured with two items (item example: “I often have to hurry and still cannot complete my work”). Scale reliability was determined with Cronbach’s α = 0.85. *Job control* was assessed with three items (e.g., “I can determine for myself how to do my work”; α = 0.86). *Social climate* was captured by two items (e.g.; “In this unit, work relationships with supervisors are based on trust”; α = 0.91). All items were answered on a five-point scale ranging from *not at all* to *to a very great extent*.

##### Technology-related work factors (“technostressors”)

For the measurement of work factors specifically related to digital technologies, we used 11 scales capturing a broad spectrum of potential technostressors:

For hindrance technostressors, four scales developed by Ragu-Nathan et al. (2008) (German translations based on Gimpel et al. (2018)) were applied: *techno-overload* (3 items; e.g., “I am forced by digital technologies to do more work than I can handle”; α = 0.84), *techno-complexity* (3 items; e.g., “I do not know enough about digital technologies to handle my job satisfactorily”; α = 0.87), *techno-uncertainty* (2 items; e.g., “There are always new developments in the digital technologies we use in our organization”; α = 0.75) and *techno-insecurity* (3 items; e.g., “I have to constantly update my skills on digital technologies to avoid being replaced”; α = 0.63). Further scales captured: *work interruptions* (3 items, adapted from Glaser et al. 2020; Büssing and Glaser 2002; e.g., “I often have to interrupt my work due to electronic messages [e.g., e-mail, device message]”; α = 0.70); *multitasking* requirements (2 items, adapted from Semmer et al. 1999; e.g., “Due to digital technologies I have to work on several tasks at the same time”; α = 0.90); and *information overload* (2 items, Piecha and Hacker 2020; e.g., “I feel that the information I receive via on-duty digital media is too much”; α = 0.93).

For challenge technostressors, the following scales were utilized: *reliability* (2 items, Ayyagari et al. 2011; Gimpel et al. 2018; e.g., “The digital technologies I use behave in a highly consistent way”; α = 0.90); *usefulness* (3 items, Ayyagari et al. 2011; Moore and Benbasat 1991; e.g., “Use of digital technologies improves the quality of my work”; α = 0.94); *involvement* (2 items, e.g.: “Our end users are consulted before the introduction of new digital technologies”; α = 0.79) and *technical support provision* (2 items, e.g.: “Our end-user help desk is easily accessible”; α = 0.89) (Ragu-Nathan et al. 2008; Gimpel et al. 2018).

#### Outcomes

##### Burnout (core and secondary symptoms)

Burnout was measured using the German translation of the Burnout Assessment Tool (BAT) with the two scales core symptoms, consisting of the subscales exhaustion and mental distance, and secondary symptoms (Schaufeli et al. 2019; Glaser and Seubert 2020). The BAT was shown to have good psychometric properties (Schaufeli et al. 2020). Core symptoms were captured by two items per subscale; a sample item for exhaustion is “At work, I feel mentally exhausted”, and for mental distance “I struggle to find any enthusiasm for my work”. A total score for burnout core symptoms was calculated for each participant based on the mean of both subscales. The reliability for this scale was α = 0.79. Secondary symptoms, i.e., psychological and psychosomatic complaints, were assessed with six items; a sample item is “I suffer from headaches”. For each participant, a mean score was computed. Scale reliability was α = 0.69. Answering options ranged from *never* to *always* on a five-point scale.

##### Low-grade inflammation: C-reactive protein

We measured high-sensitivity C-reactive Protein (hs-CRP) in participants’ capillary blood using the minimally invasive dried blood spot method (McDade et al. 2007). In short, blood drops from a prick into the participant’s fingertip with a disposable lancet were collected on filter papers. The filter paper was dried at room temperature for at least 8 h and then stored in an envelope at  – 26 °C. Hs-CRP was analyzed with a “Human C-Reactive Protein/CRP Quantikine ELISA Kit” (IBL International) in the laboratory of the Chair of Health Psychology, Friedrich-Alexander University Erlangen-Nürnberg, in Nürnberg, Germany (Becker et al. 2022c for further details). The intra-assay coefficient of variation was 4.18%. Based on established cut-offs, values below 1.0 mg/L indicate a low, between 1.0 and 3.0 mg/L an average and above 3.0 mg/L a high risk for the development of cardiovascular diseases (e.g., Pearson et al. 2003).

#### Covariates

The following variables were assessed in the questionnaire as potential covariates:

*Sociodemographic characteristics*: sex (f/m/d), age (in years).

*Health-related characteristics*: body-mass index (BMI; kg/m^2^), physical activity (“Overall, how much do you care about getting enough physical activity?”, 1 = not at all – 5 = very much), smoking (no, former, current), alcohol intake (“How often do you have a drink containing alcohol, e.g., glass of wine, beer, cocktail, liquor or liqueur?”; dichotomized at ≥ 2–3 times a week; translated, Bush et al. 1998), chronic conditions (yes, no), hormone medication (for contraception and for other reasons; only for CRP).

*Employment-related characteristics*: Shiftwork (yes, no), night shift (yes, no), profession (nurse, physician, medical(-technical) personnel, research staff, administration, other), professional tenure (in years), full-time job (yes, no), leadership responsibility (yes, no), extended vacation during the previous 4 weeks before testing (≥ 3 weeks; yes, no), caring for COVID-19 patients (yes, no).

#### Statistical analyses

For the technostressor scales, first an exploratory factor analysis (Principal Component Analysis [PCA] with varimax rotation) was conducted to (1) explore the structure of the variables, (2) examine the validity of the items for the measurement of technostressors, and (3) reduce variables for the sake of parsimony and to limit multi-collinearity (Field 2009). For the retrieved factors, mean scores were calculated. Prior to performing parametric tests, measures were checked for normal distribution. Due to positive skewness, both burnout scales (core symptoms = 0.79; secondary symptoms = 0.70) and hs-CRP-values (= 2.81) were transformed using natural logarithm. All predictor variables were centered using grand mean centering. Cronbach’s alpha (α) was calculated for the assessment of internal consistency.

After descriptive analyses, relevant confounders for burnout symptoms and CRP were identified using Pearson correlations, t-tests, and univariate variance analysis. For the analysis of our research questions, we applied linear regressions for each outcome: First, bivariate regressions for one control and one predictor variable at a time were calculated (crude model). Next, multivariate regressions for each predictor variable adjusted for all control variables were performed (model 1–7). Only control variables that showed significant associations with the outcomes in the first place were included in the regression models for reasons of parsimony. We applied the method of hierarchical regression with the identified covariates entered in the first step and each predictor (general work factors and technostressors) entered individually in the second step (research questions 1 and 2). Furthermore, in case of significant associations of technostressors with the outcomes, we additionally controlled for general psychosocial work factors (research question 3a), and if still significant, we tested for moderation effects of technostressors and general psychosocial work factors by including interaction terms into the multivariate models (research question 3b). Assumptions of regression analysis were checked using correlation matrices, variance inflation factor (VIF) values, Durbin-Watson test, histograms, and normal probability plots of residuals. Regarding multi-collinearity, correlations between predictors and covariates for the individual outcomes (burnout, core symptoms: *r* ≤ 0.55; burnout, secondary symptoms: *r* ≤ 0.56; CRP: *r* ≤ 0.30) and VIF values (burnout, core symptoms: ≤ 1.56; burnout, secondary symptoms: ≤ 1.54; CRP: ≤ 1.17), indicated no too strong relationships (Field 2009). All statistical analyses were performed using SPSS Statistics version 26 (IBM SPSS Inc., Chicago, Il, USA).

## Results

### Factorial validity of technostressors

Bartlett-Test (Chi^2^ (351) = 2925.67, *p* < 0.001) and Kaiser–Meyer–Olkin Measure of Sampling Adequacy (KMO = 0.83) indicated meritorious suitability of the variables for factor analysis (Kaiser 1970, 1974). The PCA revealed a six-factor-structure following the Kaiser criterion (Eigenvalue > 1). However, based on the scree-plot and theoretical as well as empirical considerations (Guadagnoli and Velicer 1988), we selected a four-factor solution explaining 61.3% of the total variance. Observed factors could be interpreted in line with the challenge-hindrance model: Factor I was classified as a challenge technostressor relating to usability characteristics and technical support provision (7 items, factor loadings: 0.59–0.88). The other three factors were conceptualized as hindrance technostressors: Factor II describes techno-overload and information overload due to digital technologies (5 items, factor loadings: 0.69–0.82); Factor III pertains to the complexity of digital technologies and associated perceived lack of skills (4 items, factor loadings: 0.72–0.87); and factor IV relates to work interruptions and multitasking demands in the context of digital technologies (5 items, factor loadings: 0.56–0.69). Items showing cross-loadings and/or loadings on a factor with only a few other variables were excluded from the analysis (*n* = 6 items). The factor loadings per item can be seen in Table S1 (Appendix). For the wording of the items, we refer to Ayyagari et al. (2011), Ragu-Nathan et al. (2008), and Gimpel et al. (2018) for German translations. The newly composed scales had high internal consistencies: α = 0.90 (factor I), α = 0.88 (factors II and III), and α = 0.84 (factor IV).

### Descriptive statistics

Five participants were excluded from the analyses because of CRP > 10 mg/L (*n* = 4) or due to incomplete survey responses (*n* = 1). Further, one person of diverse gender had to be excluded, because group comparisons were not possible. The final sample size was *n* = 167. The sample consisted of 125 women (74.9%), the mean age was 31.1 years (standard deviation, *SD* = 9.6, range: 17–60), and the average BMI was 24.1 (*SD* = 5.1, range: 16.6–45.6). Most participants were nurses (*n* = 46, 27.5%), followed by physicians (*n* = 33, 19.8%), and research staff (*n* = 30, 18.0%). The remaining participants were medical-technical personnel (for labs, pharmacy, etc.; *n* = 22, 13.2%), administrative staff (*n* = 10, 6.0%), and other (such as therapists, midwives, nutritionists, social workers, etc.; *n* = 24, 14.4%). The majority was working full-time (*n* = 129, 77.2%), and 69 (41.3%) were working on a shift schedule with 57 participants doing night shifts. Nineteen (11.4%) employees had leadership responsibilities and 26 (15.6%) were involved in the care of COVID-19 patients. Means and *SD*s as well as frequencies of all included variables are presented in Table 1.

**Table 1:** Descriptive statistics of covariates, predictor, and outcome variables

*Covariates*	*Mean (SD)*
Age, in years	31.1 (9.6)
BMI (kg/m^2^)	24.1 (5.1)
Physical activity ^1^	3.5 (1.0)
Professional tenure, in years	5.3 (7.7)
	*Frequencies (%)*
Smoking	
No	120 (71.9%)
Former	11 (6.6%)
Current	36 (21.6%)
Alcohol intake	
≤ 2–4 times per month	129 (77.2%)
≥ 2–3 times per week	36 (21.6%)
Chronic conditions (yes)	24 (14.4%)
Hormone medication, not for contraception (yes)	15 (9.0%)
Hormone medication, for contraception (yes)	24 (14.4%)
*Predictors* ^2^	*Mean (SD)*
Work overload	2.58 (1.16)
Job control	2.95 (1.08)
Social climate	3.74 (1.21)
C-TS: Usability & technical support	3.36 (0.99)
H-TS: Techno- & information overload	2.16 (0.89)
H-TS: Techno-complexity	1.66 (0.73)
H-TS: Interruptions & multitasking	2.60 (1.02)
*Outcomes*	*Mean (SD)*
Burnout: core symptoms ^3^	1.94 (0.74)
Burnout: secondary symptoms ^3^	1.94 (0.57)
C-reactive Protein (mg/L)	1.23 (1.64)

*C-TS *challenge technostressor, *H-TS *hindrance technostressor

^1^Scale range: 1 = not at all – 5 = very much

^2^Scale range: 1 = not at all – 5 = to a very great extent

^3^Scale range: 1 = never – 5 = always

*N* = 167

### Associations of work stressors with burnout (core and secondary symptoms)

Burnout core symptoms (exhaustion and mental distance) were significantly negatively associated with physical activity and longer vacations prior to testing (results not shown), and therefore, these variables were entered as covariates into the models. Results of the regression analyses on bivariate (crude) and multivariate (adjusted) associations of the covariates and predictors (general work factors and technostressors; models 1–11) with employees’ burnout core symptoms are presented in Table 2. Work overload was a significant predictor of burnout symptoms (Model 1: non-standardized regression coefficient *B* = 0.19, *p* < 0.001). For job control and social climate, no significant associations were observed. However, for all three hindrance technostressors, there were significant positive relationships with burnout core symptoms in crude and adjusted models (techno-/information overload: Model 5: *B* = 0.19, *p* < 0.001; techno-complexity: Model 6: *B* = 0.13, *p* < 0.001; interruptions and multitasking: Model 7: *B* = 0.12, *p* < 0.001). Moreover, techno-/information overload remained a significant predictor of burnout, when general work overload was controlled for (Model 8: *B* = 0.09, *p* = 0.005). We also tested for potential moderation effects, but the interaction between work overload and techno-/information overload was not significant. Concerning the included covariates physical activity and prior vacation, robust negative associations with burnout core symptoms were observed in the crude and adjusted models (Table 2).

**Table 2:** Crude associations of control and predictor variables as well as adjusted associations of predictor variables with burnout (core symptoms)

		BAT core symptoms													
		Crude		Model 1		Model 2		Model 3		Model 4		Model 5		Model 6	
		B	*P*-value	B	*P*-value	B	*P*-value	B	*P*-value	B	*P*-value	B	*P*-value	B	*P*-value
Covariates	Physical activity*	**− 0.11**	**<.001**	**− 0.05**	**.034**	− 0.12	**<.001**	**− 0.12**	**<.001**	**− 0.12**	**<.001**	**− 0.08**	**.003**	**− 0.10**	**.001**
	Prior vacation (no, yes)	**− 0.19**	**.004**	− 0.05	.358	**− 0.17**	**.007**	**− 0.17**	**.006**	**− 0.18**	**.005**	**− 0.12**	**.033**	**− 0.16**	**.007**
Predictors:															
General work factors	Work overload	0.21	** <.001**	0.19	**<.001**										
	Job control	0.04	.118			0.05	.099								
	Social climate	0.02	.444					0.02	.425						
Techno-stressors	C-TS: usability & technical support	0.05	.089							0.04	.188				
	H-TS: techno-/ information overload	0.23	**<.001**									**0.19**	**<.001**		
	H-TS: techno-complexity	0.16	**<.001**											**0.13**	**<.001 **
	H-TS: interruptions & multitasking	0.15	**<.001**												
	Work overload* information & techno-overload														
Model fit (R^2^)		0.00–0.41		0.45		0.15		0.13		0.15		0.32		0.20	

		BAT core symptoms									
		Model 7		Model 8		Model 9		Model 10		Model 11	
		B	*P*-value	B	*P*-value	B	*P*-value	B	*P*-value	B	*P*-value
**Covariates**	Physical activity*	**− 0.11**	**<.001**	**− 0.05**	**.047**	**− 0.05**	**.041**	**− 0.06**	**.030**	− 0.04	.099
	Prior vacation (no, yes)	**− 0.12**	**.044**	− 0.04	.434	− 0.05	.378	− 0.04	.469	− 0.03	.612
**Predictors**:											
General work factors	Work overload			**0.16**	**<.001**	**0.19**	**<.001**	**0.18**	**<.001**	**0.17**	**<.001**
	Job control										
	Social climate										
Techno-stressors	C-TS: usability & technical support										
	H-TS: techno-/ information overload			**0.09**	**.005**					**0.10**	**.002**
	H-TS: techno-complexity					0.03	.380				
	H-TS: interruptions & multitasking	**0.12**	**<.001**					0.03	.188		
	Work overload* information & techno-overload									− 0.04	.082
Model fit (R^2^)		0.24		0.47		0.45		0.45		0.49	

*B* non-standardized regression coefficient, *C-TS* Challenge technostressor, *H-TS* Hindrance technostressor, *1 = not at all – 5 = very much; N = 167; bold if p < .05

Crude: bivariate regressions (one control and one predictor variable at a time)

Model 1: control variables + work overload

Model 2: control variables + job control

Model 3: control variables + social climate

Model 4: control variables + usability & technical support

Model 5: control variables + techno-/ information overload

Model 6: control variables + techno-complexity

Model 7: control variables + interruptions & multitasking

Model 8: control variables + work overload + techno-/ information overload

Model 9: control variables + work overload + techno-complexity

Model 10: control variables + work overload + interruptions & multitasking

Model 11: control variables + work overload + techno-/ information overload + work overload* techno-/ information overload

For burnout secondary symptoms, i.e., psychological and psychosomatic complaints, participants’ sex, physical activity, smoking, and leadership responsibility were identified as relevant covariates. Results of the bivariate and multivariate regression analyses including these covariates, general work factors, and technostressors are depicted in Table 3. Again, work overload significantly predicted secondary symptoms (Model 1: *B* = 0.05, *p* = 0.014). Additionally, techno-/information overload (Model 5: *B* = 0.07, *p* = 0.004) and techno-complexity (Model 6: *B* = 0.06, *p* = 0.038) were significantly related to secondary burnout symptoms. However, when including general work overload in the models (model 8 and 9), associations of the technostressors were not statistically significant. As for the covariates, sex was a significant predictor of burnout secondary symptoms across all models, such that the female sex was associated with increased ratings. In addition, smoking was consistently a significant positive predictor for reporting secondary symptoms. On the contrary, there were trends across the models for negative associations between both physical activity (i.e., higher level of physical activity was associated with lower symptom ratings) and leadership responsibilities with secondary burnout symptoms (i.e., leaders reported less symptoms).

**Table 3:** Crude associations of control and predictor variables as well as adjusted associations of predictor variables with burnout (secondary symptoms)

		BAT secondary symptoms											
		Crude		Model 1		Model 2		Model 3		Model 4		Model 5	
		B	*P*-value	B	*P*-value	B	*P*-value	B	*P*-value	B	*P*-value	B	*P*-value
**Covariates**	Sex (male, female)	**0.20**	**<.001**	**0.13**	**.011**	**0.14**	**.007**	**0.13**	**.011**	**0.13**	**.009**	**0.14**	**.007**
	Physical activity^1^	**− 0.07**	**.003**	− 0.04	.083	**− 0.06**	**.015**	**− 0.05**	**.017**	**− 0.06**	**.015**	− 0.04	.062
	Smoking (no, former, current)	**0.07**	**.010**	**0.05**	**.046**	**0.06**	**.027**	**0.05**	**.040**	**0.06**	**.027**	**0.06**	**.022**
	Leadership responsibility (no, yes)	**− 0.19**	**.008**	− 0.13	.050	− 0.13	.063	**− 0.14**	**.045**	− 0.13	.054	− 0.13	.058
**Predictors **													
General work factors	Work overload	**0.06**	** <.001**	**0.05**	**.014**								
	Job control	− 0.03	.108			− 0.03	.092						
	Social climate	**− 0.04**	**.032**					**− **0.03	.067				
Techno-stressors	C-TS: usability & technical support	− 0.02	.344							− 0.02	.474		
	H-TS: techno-/ information overload	**0.08**	**.002**									**0.07**	**.004**
	H-TS: techno-complexity	**0.09**	**.003**										
	H-TS: interruptions & multitasking	0.01	.590										
Model fit (R^2^)		0.00–0.10		0.20		0.18		0.18		0.17		0.21	

		BAT secondary symptoms							
		Model 6		Model 7		Model 8		Model 9	
		B	*P*-value	B	*P*-value	B	*P*-value	B	*P*-value
**Covariates**	Sex (male, female)	**0.11**	**.037**	**0.13**	**.009**	**0.13**	**.008**	**0.11**	**.027**
	Physical activity^1^	**− 0.05**	.**035**	**− 0.05**	.**022**	− 0.04	.103	− 0.04	.094
	Smoking (no, former, current)	**0.06**	**.028**	**0.06**	**.023**	**0.05**	**.033**	**0.05**	**.044**
	Leadership responsibility (no, yes)	**− 0.15**	**.030**	**− 0.14**	**.036**	− 0.13	.059	**− 0.14**	**.040**
**Predictors**									
General work factors	Work overload					0.03	.266	0.04	.068
	Job control								
	Social climate								
Techno-stressors	C-TS: usability & technical support								
	H-TS: techno-/ information overload					0.05	.071		
	H-TS: techno-complexity	**0.06**	**.038**					0.04	.247
	H-TS: interruptions & multitasking			0.02	.282				
Model fit (R^2^)		0.19		0.17		0.22		0.20	

*B* non-standardized regression coefficient, *C-TS* Challenge technostressor, *H-TS* Hindrance technostressor, *1 = not at all - 5 = very much; N = 167; bold if *p* < .05

Crude: bivariate regressions (one control and one predictor variable at a time)

Model 1: control variables + work overload

Model 2: control variables + job control

Model 3: control variables + social climate

Model 4: control variables + usability & technical support

Model 5: control variables + techno-/ information overload

Model 6: control variables + techno-complexity

Model 7: control variables + interruptions & multitasking

Model 8: control variables + work overload + techno-/ information overload

Model 9: control variables + work overload + techno-complexity

### Associations of work stressors with low-grade inflammation (C-reactive protein)

Bivariate (crude) and multivariate (adjusted) regressions for the covariates, predictors (general and technostressors) and hs-CRP are presented in Table 4. Regarding relevant covariates, age (only in the crude model), BMI, use of contraceptives, and leadership responsibility were consistently positively associated with employees’ hs-CRP levels. Physical activity was negatively associated with hs-CRP (only in the crude model). For the predictors, results showed a statistical trend for a relation of work overload and hs-CRP (only in the crude model: *B* = 0.15, *p* = 0.071). For the other general work factors and the technostressors, no significant associations were observed neither in the crude nor in the adjusted models.

**Table 4:** Crude associations of control and predictor variables as well adjusted associations of predictor variables with C-reactive protein

		C-reactive protein															
		Crude		Model 1		Model 2		Model 3		Model 4		Model 5		Model 6		Model 7	
		B	*P*-value	B	*P*-value	B	*P*-value	B	*P*-value	B	*P*-value	B	*P*- value	B	*P*-value	B	*P*-value
**Covariates**	Age	**0.02**	**.044**	0.01	.315	0.01	.382	0.01	.347	0.01	.261	0.01	.303	0.01	.243	0.01	.316
	BMI	**0.10**	** <.001**	**0.11**	** <.001**	**0.11**	** <.001**	**0.11**	** <.001**	**0.11**	** <.001**	**0.11**	** <.001**	**0.11**	** <.001**	**0.11**	** <.001**
	Physical activity*	** – 0.26**	**.012**	– 0.12	.215	– 0.15	.103	– 0.15	.097	– 0.17	.076	– 0.16	.093	– 0.17	.070	– 0.16	.092
	Use of contraceptives (no, yes)	**0.80**	**.005**	**1.07**	** <.001**	**1.11**	** <.001**	**1.11**	** <.001**	**1.12**	** <.001**	**1.12**	** <.001**	**1.14**	** <.001**	**1.12**	** <.001**
	Leadership responsibility (no, yes)	**0.77**	**.015**	**0.55**	**.042**	0.51	.066	**0.54**	**.048**	**0.57**	**.040**	**0.55**	**.049**	0.54	.050	0.54	.052
**Predictors:**																	
General work factors	Work overload	0.15	.071	0.12	.136												
	Job control	0.05	.582			0.08	.318										
	Social climate	– 0.09	.298					– 0.06	.386								
Techno-stressors	C-TS: usability & technical support	– 0.07	.508							– 0.08	.386						
	H-TS: techno-/ information overload	0.08	.463									0.01	.928				
	H-TS: techno-complexity	0.04	.799											– 0.09	.486		
	H-TS: interruptions and multitasking	0.13	.190													0.03	.707
Model fit (R^2^)		0.00–0.16		0.29		0.28		0.28		0.29		0.28		0.29		0.28	

*B* non-standardized regression coefficient, *C-TS* challenge technostressor, *H-TS* hindrance technostressor, *1 = not at all - 5 = very much; bold if *p* < .05

Crude: bivariate regressions (one control and one predictor variable at a time)

Model 1: control variables + work overload

Model 2: control variables + job control

Model 3: control variables + social climate

Model 4: control variables + usability & technical support

Model 5: control variables + techno-/ information overload

Model 6: control variables + techno-complexity

Model 7: control variables + interruptions & multitasking

## Discussion

The aim of this study was to assess associations of technostressors at work with psychological (i.e., burnout symptoms) and biological (i.e., hs-CRP as an inflammatory marker) health outcomes. To the best of our knowledge, this is the first study to investigate the potential effects of technostressors on immune activity in terms of chronic low-grade inflammation. Research on technostress as a risk factor for adverse psychophysiological health is still “work-in-progress” and there is a broad range of different theoretical terms and measures largely due to the interdisciplinary character of research on this phenomenon (Dragano and Lunau 2020, p. 411). With the ever-increasing digitalization and the ubiquity of digital technologies in employees’ workplaces, it is timely to advance our understanding of the phenomenology of technostress and the consequences for employee health, both positive and negative.

To this end, our research approach comprised two steps: We measured technostress with a comprehensive questionnaire including 27 items from 11 scales based on the literature. In an attempt to identify underlying, latent dimensions within this compilation of variables, we first conducted an exploratory factor analysis (Field 2009). We extracted four factors and interpreted them in line with the challenge-hindrance model (Califf and Sarker 2020; Tarafdar et al. 2019). Factor I—the challenge technostressor “usability and technical support”—reflects the positive aspect of technostress, i.e., technology characteristics appraised as beneficial for work-related achievement (Podsakoff et al. 2007; Califf and Sarker 2020). This factor includes reliability of digital technologies, their usefulness for the execution of job tasks, and technical support provision at work. Factors II-IV represent hindrance technostressors, i.e., stressors appraised as thwarting job-related accomplishment (Podsakoff et al. 2007). Factor II (“techno- and information overload”) can be interpreted as an extension of the well-established stressor techno-overload (Tarafdar et al. 2007), i.e., increased workload and work pace due to ICTs, by information overload, i.e., the feeling of too much information (“information flood”) transmitted through ICTs (Piecha and Hacker 2020). Factor III (“techno-complexity and lack of skills”) describes the users’ feeling of inadequacy regarding their skills due to high complexity of ICTs requiring extra effort; this is accompanied by the feeling of pressure through coworkers with better ICT knowledge and skills (Tarafdar et al. 2007). And lastly, factor IV (“interruptions and multitasking”) represents frequent interruptions of the workflow due to digital technologies and the requirement to perform several tasks simultaneously or alternately (i.e., multitasking) (Baethge and Rigotti 2013, 2010). As a second step, we investigated associations of these four factors with employees’ burnout symptoms and low-grade inflammation under consideration of other job characteristics (work overload, control, social climate) and a broad range of potential confounders. Regarding our research questions, we yielded the following results:

First, we found associations of hindrance technostressors and burnout symptoms. In particular, techno-/information overload, techno-complexity as well as interruptions and multitasking were positively related to core symptoms of burnout. Moreover, techno-/information overload and techno-complexity were associated with secondary burnout symptoms. Our results thus add to the preliminary evidence for a positive association of technostressors and burnout (Dragano and Lunau 2020; Berg-Beckhoff et al. 2017). A prior study showed that high quantity and poor quality (i.e., high ambiguity) of workplace e-mail contributed to emotional exhaustion (Brown et al. 2014). E-mail stressors can be regarded as manifestations of our identified dimensions techno-/information overload, in terms of overstraining users’ information-processing capacity (Eppler and Mengis 2004), and interruptions/multitasking by causing immediate interruptions of the workflow and the perceived requirement to perform several tasks simultaneously, in order to manage the amount of emails. Concerning techno-complexity, however, other studies did not find effects on burnout, but—similar to our results—effects of techno-overload and techno-insecurity (Califf and Brooks 2020; Day et al. 2012). With regard to secondary burnout symptoms, our observations are consistent with a previous investigation showing associations of telecommunication system engineers’ perceived mental workload and lack of skills with psychosomatic symptoms such as headache, mental fatigue, or restlessness (Arnetz and Wiholm 1997). Altogether, our observations call for a more nuanced picture with potentially differential effects of distinct technostressors on various aspects of burnout.

Second, even after adjusting for work overload, techno-/information overload still significantly predicted burnout core symptoms and also secondary symptoms on a trend level. In contrast to previous studies (Califf and Sarker 2020; Ayyagari et al. 2011), we did not find any associations of the challenge technostressor with our outcomes, i.e., no direct health-promoting effects. Nonetheless, we observed a small negative effect of social climate on secondary burnout symptoms, in that good social climate was related to fewer symptoms. Drawing upon the buffer hypothesis of the JDC model (Karasek 1979; van der Doef and Maes 1999), we sought to identify interaction effects between the job characteristics, i.e., whether job control, social climate or the challenge technostressor reduces the potential associations of work overload and the hindrance technostressors with the outcomes. We did not detect any interactions of technostressors and general work stressors. This is in line with a current review suggesting strong evidence for the absence of the theorized interaction effect between job demands and control in the prediction of workers’ well-being (Huth and Chung-Yan 2022).

Third, we did not observe associations of technostressors with low-grade inflammation (hs-CRP). We just observed one, yet non-significant association of work overload in the crude model. This preliminary finding adds to the research base on the JDC(S) model and inflammatory markers, which heretofore is limited and inconclusive (Kaltenegger et al. 2021; Wright et al. 2020; Nakata 2012). Again, we could not identify any effects of job control and social climate on hs-CRP, whereas few previous investigations reported protective effects of job resources such as supervisor support (Eguchi et al. 2016), control (Shirom et al. 2008) or organizational justice (Elovainio et al. 2010) in terms of reduced inflammation. In hospital employees, respective investigations are sparse. One recent study surprisingly found a positive relationship of job autonomy and CRP among geriatric care professionals, perhaps due to greater responsibilities and experiences of excessive demands (Kaltenegger et al. 2022).

With regard to the included covariates, physical activity was consistently negatively associated with burnout symptoms and hs-CRP (significantly only in the crude model). While it is well-documented that physical activity during leisure time has beneficial effects on physical and mental health, occupational physical activity can be detrimental—a phenomenon called the physical activity health paradox (Holtermann et al. 2012; Lee et al. 2021). This aspect deserves careful consideration especially in the healthcare sector, where many professions face high physical demands such as lifting heavy loads, working in awkward postures, or walking long distances. Interestingly, participants in leadership positions had higher levels of CRP but reported less secondary burnout symptoms. Although higher occupational position has been associated with lower inflammation (e.g., Fraga et al. 2015), one can speculate that this small group of employees with leadership responsibilities at a large university hospital might be exposed to a particularly high work stress level and that confounding factors, such as profession, sex, age and professional tenure might explain this observation.

In sum, our results suggest that technostress in the form of techno- and information overload is associated with burnout symptoms. The association remained significant when work overload was included in the multivariate model. This finding indicates that (information) overload caused by digital technology use is a distinct work stressor with genuine consequences for psychological health. However, these might not be “strong” enough to manifest on a biological level in terms of chronic physiological activity, such as low-grade inflammation.

### Limitations

Some important limitations need to be considered when interpreting our results. First, this study is cross-sectional and, therefore, no inferences concerning causality can be drawn. Second, based on the a-priori power analysis for the complete prospective cohort study yielding a required sample size of *N* = 200, our sample size may be regarded as too small and hence, our study might have been underpowered. However, as this sample consists of new employees, for a valid assessment of their work situation and associated influences, we rigorously had to exclude a large amount of the original sample. Participants who had not started their job at the university hospital at the time of examination and who were not working prior to the start of employment (because of studies/school, parental leave, unemployment or similar) were not included. Nonetheless, the heterogeneity in participants’ life and work situations remains a critical issue. Therefore, we sought to control for potentially influencing factors, such as professional tenure and long vacation or leave in the weeks before testing. Due to the specific sampling procedure and the strict exclusion criteria, our sample consisted mainly of healthy participants of rather young age and short professional tenure, potentially resulting in a floor effect in terms of chronic stress experience. This might explain the comparatively low values in the burnout scales. However, the mean hs-CRP level was in the range of average risk for cardiovascular disease (Pearson et al. 2003). Participants’ age might have also played a role in the evaluation of technostressors, as age has been identified as an important moderator (Reinecke et al. 2017; Tams et al. 2014). In sum, our recruitment method (i.e., pre-employment medical check) may have introduced bias concerning the sample and associations. The cohort was younger compared to the average healthcare worker, what might limit the external validity of our results. We checked for associations of participants’ professions with the outcomes and did not find any significant differences. Therefore, we did not include profession as a covariate in our analyses. It can be assumed that most of the jobs at this large university hospital were affected by the ever-increasing computerization, both in direct (such as medical care) and indirect clinical work (such as administration and research). Nonetheless, different professions might have been affected differently by technology exposure and inherent technostressors. Future research should hence distinguish between professional groups more clearly, in order to identify groups at particular risk for technostress, for instance due to a lack of digital competence (Golz et al. 2021). Further limitations pertain to the measurement of our outcomes: Burnout core symptoms were measured with only two subscales of the BAT with just few items; only hs-CRP concentrations were utilized as an inflammatory marker, while there a many other indicators of low-grade inflammation, such as cytokines (Kaltenegger et al. 2020, for a list). Although we collected broad screening information, we acknowledge that several, potentially confounding lifestyle and behavioral factors were not measured in sufficient detail, such as step count or weight change. Moreover, the inclusion of additional biomarkers of other stress systems, such as ANS (e.g., heart rate [variability]) and HPA-axis (e.g., cortisol), would be promising for a more comprehensive picture and deeper understanding of the linkage of (techno-)stress, biomarkers and burnout.

### Implications for further research and occupational practice

Given that research on psychophysiological effects of technostressors is scarce, our results should be considered preliminary until further investigations can replicate them. Nonetheless, our study provides valuable methodological implications for future research. In particular, we suggest the following avenues with regard to design, measures, and samples: First, prospective studies are needed for a deeper understanding of dynamic and causal processes. Full-panel designs where each predictor and outcome variable is assessed at all measurement time points are suitable to identify both normal (i.e., stressor-to-strain) and reversed (i.e., strain-to-stressor) effects (Taris and Kompier 2014). Second, our operationalization of technostressors and the four-factor-structure should be scrutinized in future studies, and beyond the commonly studied negative aspects of technostress (i.e., hindrance technostressors), also positive (i.e., challenge technostressors) should be taken into account. Moreover, it is crucial to apply multiple methods, i.e., a combination of self-report data with measurable markers for biological stress, especially for chronic stress given its key role in long-term health. There is a long-standing debate on viable approaches to measure work-related stress (Semmer et al. 2003). The inclusion of biomarkers as outcome variables overcomes the problem of common method variance when both predictor and outcome variables are measured with self-report (Semmer et al. 2003). Moreover, self-report can be biased by individual response tendencies, whereas physiological data are less easily influenced by the participant or the examiners’ expectations. However, also biomarkers have been discussed regarding conceptual, such as ambiguities in interpretation, as well as methodological issues, including limited reliability and potential confounding influences. Thus, self-report should not just be replaced—instead for an optimal assessment of psychobiological effects of work stress, a combination of various methods and multiple information sources is desirable (Semmer et al. 2003). Lastly, more research on technostress in hospital employees is necessary against the backdrop of the vast implementation of health information technology in hospitals.

For occupational health and safety management, there have been calls to consider job stressors related to the digitalization of work in the psychosocial risk assessment (Diebig et al. 2018; Chiappetta 2017). This will facilitate effective prevention and intervention measures on an organizational/structural as well as individual/behavioral level. Several strategies to cope with technostress have been described by healthcare managers, referring to establishing norms, such as good email culture, individual resources, such as digital literacy, and organizational resources, such as accessible and efficient IT support (Stadin et al. 2020). However, there is a lack of systematic prevention and intervention studies on work-related technostress (Rohwer et al. 2022). In general, workplace physical exercise interventions have been proven useful in the reduction of low-grade inflammation (Kaltenegger et al. 2021) and burnout (i.e., exhaustion) (Naczenski et al. 2017).

### Conclusions

To the best of our knowledge, this is the first study on technology-related stress at work and chronic low-grade inflammation. Low-grade inflammation is a key pathway through which stress “gets under the skin” and ultimately affects humans’ health. However, biological effects of technostress have been under-researched. We did not find associations of technostressors with inflammation, but techno- and information overload was consistently associated with burnout symptoms in employees of a university hospital. Nevertheless, due to peculiarities of our sample we cannot negate additional biological effects of this stressor in general and deem future research on this question as highly necessary.

## Supplementary Information

Below is the link to the electronic supplementary material.

Supplementary file1 (DOCX 19 KB)

## Data Availability

The dataset generated during and/or analyzed during the current study are not publicly available due to them containing information that could compromise research participant privacy/consent. Public data availability was not considered in the application for ethical approval. However, upon reasonable request data are available from the corresponding author (HK).
