# Supplementary material for: Associations of technostressors at work with burnout symptoms and chronic low-grade inflammation: a cross-sectional analysis in hospital employees
Source: Int Arch Occup Environ Health. 2023 May 6;96(6):839–56. doi: 10.1007/s00420-023-01967-8 (PMC10163295; doi:10.1007/s00420-023-01967-8)
Supplement: Supplementary file 1 — Supplementary file1 (DOCX 19 KB) [file 420_2023_1967_MOESM1_ESM.docx]

**Appendix**

**Table S1** Results of the Principal Component Analysis: Factor loadings per item

| **Item** | **Factor I** Challenge technostressor: Usability and technical support | **Factor II**  Hindrance technostressor: Techno- and information overload | **Factor III**  Hindrance technostressor:  Techno-complexity and lack of skills | **Factor IV**  Hindrance technostressor: Interruptions and multitasking |
| --- | --- | --- | --- | --- |
| C-TS-reliab2 | .88 | .18 | .02 | -.05 |
| C-TS-reliab1 | .85 | .21 | -.02 | -11 |
| C-TS-useful1 | .76 | -.02 | -.01 | .15 |
| C-TS-useful2 | .75 | -.06 | -.05 | .19 |
| C-TS-useful3 | .74 | -.04 | -.01 | -.20 |
| C-TS-tecsup2 | .68 | -.07 | .29 | .36 |
| C-TS-tecsup1 | .59 | .03 | .27 | .45 |
| H-TS-tecoverl1 | .09 | .82 | .23 | .14 |
| H-TS-infoverl2 | -.02 | .78 | .17 | .22 |
| H-TS-infoverl1 | .06 | .75 | .17 | .24 |
| H-TS-tecoverl2 | .06 | .71 | .19 | .24 |
| H-TS-tecoverl3 | .11 | .69 | .25 | .14 |
| H-TS-teccomp2 | .12 | .14 | .87 | .04 |
| H-TS-teccomp1 | .05 | .16 | .85 | .15 |
| H-TS-teccomp3 | -.01 | .23 | .83 | -.01 |
| H-TS-tecinsec3 | -.02 | .23 | .72 | .12 |
| H-TS-interrup2 | .23 | .13 | .09 | .69 |
| H-TS-interrup1 | .10 | .45 | .09 | .69 |
| H-TS-interrup3 | -.03 | .16 | .06 | .67 |
| H-TS-multita1 | .20 | .38 | .07 | .58 |
| H-TS-multita2 | .19 | .44 | .04 | .56 |
| CT-TS-involv1 | .19 | .07 | .10 | .10 |
| CT-TS-involv2 | .32 | .05 | .03 | .16 |
| H-TS- tecinsec1 | -.17 | .42 | .18 | -.04 |
| H-TS- tecinsec2 | .15 | .10 | .45 | .09 |
| H-TS- uncert1 | .22 | .26 | .08 | .11 |
| H-TS- uncert2 | .06 | .09 | .04 | .24 |

*Note*. C-TS = challenge technostressor; H-TS = hindrance technostressor; shaded in grey: those items built one factor/ scale; grey font: items were discarded
